# Astrocytic Ca^2+^ Signaling in Epilepsy

**DOI:** 10.3389/fncel.2021.695380

**Published:** 2021-07-15

**Authors:** Kjell Heuser, Rune Enger

**Affiliations:** ^1^Department of Neurology, Oslo University Hospital, Rikshospitalet, Oslo, Norway; ^2^Letten Centre and GliaLab, Division of Anatomy, Department of Molecular Medicine, Institute of Basic Medical Sciences, University of Oslo, Oslo, Norway

**Keywords:** astrocyte, epilepsy, calcium signaling, IP3, epileptogenesis, ictogenesis, astrogliosis

## Abstract

Epilepsy is one of the most common neurological disorders – estimated to affect at least 65 million worldwide. Most of the epilepsy research has so far focused on how to dampen neuronal discharges and to explain how changes in intrinsic neuronal activity or network function cause seizures. As a result, pharmacological therapy has largely been limited to symptomatic treatment targeted at neurons. Given the expanding spectrum of functions ascribed to the non-neuronal constituents of the brain, in both physiological brain function and in brain disorders, it is natural to closely consider the roles of astrocytes in epilepsy. It is now widely accepted that astrocytes are key controllers of the composition of the extracellular fluids, and may directly interact with neurons by releasing gliotransmitters. A central tenet is that astrocytic intracellular Ca^2+^ signals promote release of such signaling substances, either through synaptic or non-synaptic mechanisms. Accruing evidence suggests that astrocytic Ca^2+^ signals play important roles in both seizures and epilepsy, and this review aims to highlight the current knowledge of the roles of this central astrocytic signaling mechanism in ictogenesis and epileptogenesis.

## Introduction

Epilepsy is one of the most common neurological disorders – estimated to affect around 1% of the world’s population (Hesdorffer et al., 2011; Neligan et al., 2012; Beghi, 2016). It is a chronic disorder, characterized by sudden, violent perturbations of normal brain function, causing social stigma, morbidity, and risk of premature death. In spite of a multitude of drugs for the treatment of epilepsy, about 30% of patients are not able to control their seizures with seizure suppressing medication (French, 2007; Perucca and Gilliam, 2012).

There is a striking lack of knowledge of the pathophysiological cellular mechanisms at play in epilepsy. For instance, the process transforming normal brain matter to a focus for epileptic seizures – the process of epileptogenesis – is not well understood. Also, the central question of what sets in motion an epileptic seizure – ictogenesis – remains unanswered. Most of the epilepsy research has so far focused on how to dampen neuronal discharges and to explain how changes in intrinsic neuronal activity or neuronal network function cause seizures. As a result, pharmacological therapy has been limited to symptomatic treatment aiming at neuronal targets. Given the expanding spectrum of roles ascribed to the non-neuronal constituents of the brain, it is natural to take a closer look at astrocytes as potential targets for epilepsy treatment.

Astrocytes are critical homeostatic controllers of extracellular glutamate and K^+^ levels (Rothstein et al., 1996; Larsen et al., 2014; Danbolt et al., 2016). Numerous studies have also demonstrated that astrocytes have important roles in supporting the neurons metabolically (Pellerin and Magistretti, 1994; Lundgaard et al., 2015) and that they have the capability of altering the vascular tone (Mulligan and MacVicar, 2004; Haydon and Carmignoto, 2006; Gordon et al., 2008). Increasing evidence suggests that astrocytes play important roles in brain state transitions and maintenance (Paukert et al., 2014; Poskanzer and Yuste, 2016; Szabó et al., 2017; Bojarskaite et al., 2020). Notably, astrocytes seem to also directly partake in brain signaling by releasing substances that affect neurons at the so-called tripartite synapse (Perea et al., 2009; Bindocci et al., 2017; Martin-Fernandez et al., 2017). A central tenet is that astroglial intracellular Ca^2+^ signals promote such “gliotransmitter” release, either through synaptic or non-synaptic mechanisms (Perea et al., 2014; Bazargani and Attwell, 2016). Glutamate, purines and D-serine are examples of transmitter substances that are thought to be released from astrocytes in a Ca^2+^ dependent manner (ibid.).

Perturbation of astrocytic Ca^2+^ signaling has been demonstrated in seizures and in epileptic tissue, potentially affecting both the homeostatic functions and signaling functions of astrocytes. These downstream mechanisms are largely speculative in the context of epilepsy but reflect the knowledge of roles of astrocytic Ca^2+^ signaling in physiology. Here, we discuss the relatively limited body of studies directly assessing astrocytic Ca^2+^ signaling in epilepsy, and briefly discuss potential downstream effects (Table 1). For the sake of structure and simplification, we arrange the topic into paragraphs on ictogenesis (i.e., the emergence of seizure activity), and epileptogenesis (i.e., the process by which the brain develops the predisposition of generating spontaneous seizures). These two processes are highly interconnected (Blauwblomme et al., 2014), but animal studies are often designed to study one of these two facets of epilepsy, and hence provide a framework for the further discussion.

**TABLE 1:** Key publications investigating the roles of astrocytic Ca^2 +^ signalling in ictogenesis and epileptogenesis.

Publication	Model	Ca^2+^ indicator	Main findings
**Astrocytic Ca^2^**^+^ **signaling in ictogenesis**			
Kang et al., 2005	Rat hippocampal slices, 4-AP	Fluo-4 AM	Adding IP_3_ in astrocytes causes epileptiform activity due to glutamate, and that astrocytic Ca^2+^ signals occur during 4-AP seizures
Tian et al., 2005	Rat hippocampal slices: 4-AP, zero-Mg^2+^, bicuculline, penicillin Mouse cortex, *in vivo*, anesthetized: local injection of 4-AP	Fluo-4 AM	Increased astrocytic Ca^2+^ signaling *in vivo* during spread of 4-AP seizures, as well as showing that uncaging Ca^2+^ in astrocytes and extrasynaptic sources of glutamate triggered paroxysmal depolarization shifts
Fellin et al., 2006	Mouse cortical-hippocampal slices: zero-Mg^2+^ and picrotoxin, or 0.5 mM Mg^2+^ and 8.5 mM K^+^	Indo-1 AM or OGB-1 AM	A correlation between astrocytic Ca^2+^ and SICs, but activation of extrasynaptic NMDA activation by astrocytes is not necessary for either ictal or interictal epileptiform events
Ding et al., 2007	Mouse, *in vivo*, anesthetized. Pilocarpine s.c., 350 mg/kg	Fluo-4 AM	Increase in astrocytic Ca^2+^ signals during SE. See also under “Epileptogenesis”
Gómez-Gonzalo et al., 2010	Mouse entorhinal cortex slice: Picrotoxin/zero-Mg^2+^ Whole guinea pig: Bicuculline	OGB-1 AM / Rhod-2	Astrocytic Ca^2+^ signals are triggered by ictal but not interictal events, and can be inhibited by blocking mGluRs and purinergic receptors. Astrocytic Ca^2+^ signals contribute to the excitation of neurons, and blocking of early ictal astrocytic Ca^2+^ signals prevent spread of ictal activity.
Baird-Daniel et al., 2017	Rat cortex, *in vivo*, anesthetized. 4-AP. Blocking astrocytic Ca^2+^ signals and gap junctions with fluoroacetate and carbenoxolone, respectively	OGB-1 AM or Rhod-2 AM	Increased Ca^2+^ signals in astrocytes during seizures, but blocking of these did not affect epileptiform discharges or vascular dynamics associated with the seizures
Heuser et al., 2018	Mouse hippocampus, *in vivo*, unanesthetized, “dual color” Ca^2+^ imaging of hippocampal neurons and astrocytes	GCaMP6f in astrocytes	Prominent astrocytic Ca^2+^ activity preceding local neuronal recruitment to seizure activity in hippocampus
Diaz Verdugo et al., 2019	Zebra fish: PTZ	GCaMP6s in astrocytes	Large activations of astrocytic Ca^2+^ signals in the pre-ictal state and that astrocytic Ca^2+^ signals contribute to excitation of neurons
Zhang et al., 2019	Mouse cortex, *in vivo*, anesthetized: local injection of 4-AP	OGB-1 AM	Absolute levels of Ca^2+^ in the astrocytic endfeet correlates with vascular tone during seizures

**Astrocytic calcium signaling in epileptogenesis**			

Ding et al., 2007	Mouse cortex, *in vivo*, anesthetized: Pilocarpine s.c. 350 mg/kg. 3D post SE	Fluo-4 AM	An increase in astrocytic Ca^2+^ signals at day 3 after SE due to mGluR5 signaling. Blocking this hyperactivity attenuated neuronal death
Szokol et al., 2015	Mouse hippocampal slices: intracortical kainate injection. Early epileptogenesis (1, 3, and 7 days after SE)	GCaMP5E	Increased Ca^2+^ signaling in hippocampal astrocytes upon schaffer collateral stimulation at days 1 and 3 after SE mediated by mGluR
Umpierre et al., 2019	Mouse hippocampal slices, at 1–3, 7–9, or 28–30 days after SE	GCaMP5G	mGluR5-mediated Ca^2+^ signaling re-emerges in epileptogenesis
Mentioned in Shigetomi et al. (2019): Sato et al.: unpublished report	4 weeks after pilocarpine induced SE	Not known	Increased Ca^2+^ signaling in reactive astrocytes
Enger et al., 2015 conference proceedings, American Epilepsy Society conference	Mouse hippocampus, *in vivo*, unanesthetized. Chronic MTLE model of deep cortical kainate injection, imaging at 3 months after SE	GCaMP6f	Episodic spontaneous hyperactivity of reactive astrocytes within/close to the sclerotic hippocampus
Plata et al., 2018	Rat, hippocampal slices, Lithium-pilocarpine	OGB-1 AM	A reduction in large size astrocytic Ca^2+^ events in atrophic astrocytes

## Astrocytic Ca^2+^ Signaling and Ictogenesis

**FIGURE 1:**
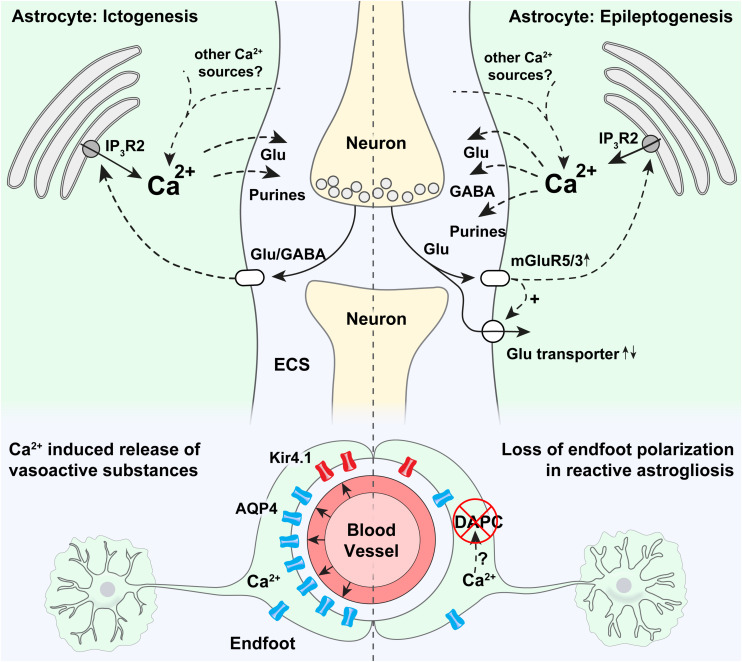
Potential roles of astrocytic Ca^2+^ signaling in epilepsy. Strong astrocytic Ca^2+^ signals have been shown to occur in the emergency of acute seizures (in ictogenesis), that are probably triggered by neurotransmitters released by neurons. Ca^2+^ increases at the onset of seizures are known to be partly mediated by release through IP_3_R2 from the endoplasmic reticulum, even though pronounced Ca^2+^ signaling is present also in mice devoid of IP_3_R2. It is thought that intracellular Ca^2+^ increases may trigger proconvulsive gliotransmitter release. In astrocytic endfeet, increased Ca^2+^ signaling has been shown to correlate with ictal vasodilation. Epileptogenesis triggers a pronounced increase in mGluR5 expression, mGluR5-mediated Ca^2+^ signaling, and increased glutamate uptake. An increase in astrocytic Ca^2+^ signaling has been demonstrated in the days after status epilepticus, and aberrant Ca^2+^ signaling at later time points in the epileptogenesis has been anecdotally reported. Increased Ca^2+^ signaling could potentially cause both the release of glutamate (pro-convulsive), purines (pro-convulsive), and GABA (anti-convulsive, through Bestrophin-1 channels). In astrocytic endfeet in epileptic tissue a pronounced loss of aquaporin-4 (AQP4) and the K^+^ inwardly rectifying channel Kir4.1 can potentially be due to Ca^2+^ activated proteases causing a disassembly of the dystrophin associated protein complex (DAPC) tethering AQP4 and Kir4.1 to perivascular endfeet.

Ictogenesis describes the emergence of seizure activity (Blauwblomme et al., 2014). The interaction between astrocytes and neurons in ictogenesis has only sparsely been investigated and findings are to some extent ambiguous or contradictory, potentially due to different experimental models (Table 1; Tian et al., 2005; Fellin et al., 2006; Gómez-Gonzalo et al., 2010; Baird-Daniel et al., 2017; Heuser et al., 2018; Diaz Verdugo et al., 2019). Astrocytes express a plethora of functionally important receptors, transporters and channels, and a role of these cells in ictogenesis is highly suggestive (Agulhon et al., 2008; Patel et al., 2019; Caudal et al., 2020). Several known astrocyte-neuron interactions involving Ca^2+^ signaling can partake in ictogenesis or in the maintenance of hypersynchronous neuronal activity, possibly by creating excitatory feedback loops (Figure 1; Gómez-Gonzalo et al., 2010; Henneberger, 2017).

Building upon seminal studies demonstrating that astrocytes are able to directly interact with neurons (Nedergaard, 1994; Parpura et al., 1994; Araque et al., 1998; Parpura and Haydon, 2000; Parri et al., 2001; Angulo et al., 2004), Fellin et al. (2006), found that eliciting astrocytic Ca^2+^ signals by photolysis of caged Ca^2 +^ and by application of ATP agonist and mGluR5 agonist triggered slow inward currents (SICs) in nearby neurons that were unaffected by application of the neuronal sodium channel blocker tetrodotoxin (Fellin et al., 2004). Soon thereafter, Tian et al. (2005) demonstrated that Ca^2+^ mediated glutamate release from astrocytes during experimentally induced seizure activity triggered slow inward currents (SICs) in neurons. These findings proposed a role for astrocytes in synchronizing neuronal activity and contributing to seizure generation (Tian et al., 2005). Further exploring which astrocytic Ca^2+^ signaling mechanisms were involved in this context, Kang et al. applied IP_3_ into astrocytes of the CA1 hippocampal region in rats, and were able to trigger epileptiform discharges in adjacent neurons (Kang et al., 2005). Later, Ding et al. (2007) were able to demonstrate increased astrocytic Ca^2+^ signaling in an *in vivo* pilocarpine epilepsy model. They proposed that this increase in Ca^2+^ signaling was due to activation of astrocytic metabotropic glutamate receptors, and that this activation led to the release of glutamate from astrocytes that could contribute to neuronal SICs through the activation of extrasynaptic neuronal NMDA receptors. By applying simultaneous patch-clamp recordings and Ca^2+^ imaging in cortical slices of the rat entorhinal cortex, Gómez-Gonzalo et al. (2010) found that Ca^2+^ elevations in astrocytes correlate with initiation and maintenance of focal seizure-like discharges, and postulated a recurrent excitatory loop between neurons and astrocytes in ictogenesis, where astrocytes play a role in recruiting neurons to ictal events, possibly through the release of gliotransmitters (Gómez-Gonzalo et al., 2010).

By using two-photon microscopy and simultaneous astrocyte and neuron Ca^2+^ imaging in the hippocampal CA1 region of awake mice, we were able to show that prominent astrocytic Ca^2+^ transients preceded local hypersynchronous neuronal activity in the emergence of kainate induced generalized epileptic seizures (Heuser et al., 2018). These findings were in agreement with the earlier results from the study of Tian et al. (2005), who also observed stereotypical astrocytic Ca^2+^ signals typically preceding local neurons in the spread of cortical seizure activity. A later work by Diaz Verdugo et al. (2019) similarly demonstrated large and synchronized astrocytic Ca^2+^ signals preceding ictal onset in zebrafish, and proposed that this signaling modulated neural excitation through glutamate release, by gap junction dependent mechanisms. In another *in vivo* study, Zhang et al. (2019), provided evidence, although correlative, that increased Ca^2+^ concentration in astrocytic endfeet governed precapillary arteriole dilation during epileptic events, suggesting a role for astrocytes in the metabolic support of neurons in seizures. In contrast to these previously mentioned studies, data from another model for focal neocortical seizures in anesthetized rats using bulk-loaded synthetic Ca^2+^ indicators found the astrocytic Ca^2+^ activation to lag behind neuronal activation and to be unnecessary for ictogenesis and the accompanying vascular dynamics (Baird-Daniel et al., 2017).

An extensive array of stimuli and corresponding signaling pathways have been shown to trigger intracellular Ca^2+^ signals in astrocytes (Zhang et al., 2019; Caudal et al., 2020). To discuss all of them would go beyond the scope of this review. One important pathway is mediated by the Inositol 1,4,5-trisphosphate (IP_3_) receptor in the endoplasmic reticulum, of which the isoform 2 (IP_3_R2) is thought to be the key functional IP_3_ receptor in astrocytes (Figure 1; Sharp et al., 1999; Parri and Crunelli, 2003; Volterra and Steinhäuser, 2004; Scemes and Giaume, 2006; Foskett et al., 2007). Lack of IP_3_R2 has been shown to abolish a large proportion of astrocytic Ca^2+^ signals (Petravicz et al., 2008; Guerra-Gomes et al., 2020). In spite of the importance of IP_3_ as a second messenger involved in astrocytic Ca^2+^ dynamics, mice lacking this receptor are overtly normal (Petravicz et al., 2008). Accordingly, studies have questioned the physiological importance of IP_3_-mediated astrocytic Ca^2+^ signaling, by for instance demonstrating normal synaptic transmission and plasticity in mice devoid of IP_3_R2 (Agulhon et al., 2010; Nizar et al., 2013; Petravicz et al., 2014). Conversely, we have demonstrated attenuated seizure activity in mice devoid of IP_3_R2 compared to WT mice following low dose intraperitoneal kainate, suggesting a proconvulsant role of astrocytic IP_3_R2 mediated Ca^2+^ elevations (Heuser et al., 2018). However, seizure activity in this study was only collected for 1 h after initiation of seizures, encouraging further investigation of the role of IP_3_R2 at later time points during epileptogenesis and in chronic epilepsy. Interestingly, even though a sizable amount of Ca^2+^ signals were still present in the knockout mice, we found that the early activation of astrocytic Ca^2+^ signals in the emergence of seizures, as discussed above, was dependent on IP_3_R2 (Heuser et al., 2018). These two observations underscore the potential importance of IP_3_R2 in ictogenesis.

Another pathway involved in astrocytic Ca^2+^ signaling attracting increasing attention for a role in epilepsy is glial purinergic signaling (Ding et al., 2007; Wellmann et al., 2018; Alves et al., 2019; Nikolic et al., 2020). Activation of astrocytic purinergic receptors triggers intracellular Ca^2+^ signals that could promote astrocytic release of gliotransmitters like glutamate or ATP, which acts on neurons and modulates excitation [reviewed in Nikolic et al. (2020)]. Importantly, Nikolic et al. (2018) provided evidence for TNFα-driven autocrine astrocyte purinergic signaling as a trigger of glutamatergic gliotransmission in a model of mesial temporal lobe epilepsy (mTLE), highlighting the complex interplay between astrocytes and microglia in epilepsy pathogenesis, discussed elsewhere (Bedner and Steinhäuser, 2019).

Most of the studies above explored the role for astrocytic Ca^2+^ signals in seizures in relation to gliotransmission, i.e., that astrocytes release transmitters that directly signal to neurons. A growing body of evidence suggests that astrocytic Ca^2+^ signals also play important roles in the control of the homeostatic functions of astrocytes. For instance they have been shown to be involved in the uptake of extracellular K^+^ through modulation of the Na^+^/K^+^ ATPase, and through the breakdown of glycogen (Wang et al., 2012; Müller et al., 2014). These mechanisms remain poorly explored in the context of epilepsy but could be important downstream effects of astrocytic Ca^2+^ signaling.

## Astrocytic Ca^2+^ Signaling and Epileptogenesis

Epileptogenesis refers to the gradual process by which a normal brain develops a propensity for recurrent seizure activity. A range of pathophysiological changes have been shown to occur during epileptogenesis, including inflammation, neurodegeneration, aberrant neurogenesis and dendritic plasticity, impaired blood-brain-barrier, epigenetic changes and alterations of the molecular composition and function of ion channels, receptors and transporters, and more (van Vliet et al., 2007; Vezzani et al., 2011; Steinhäuser and Seifert, 2012; Dingledine et al., 2014; Jessberger and Parent, 2015; Hauser et al., 2018; Escartin et al., 2021).

A common denominator of astrocytic pathophysiology associated with epileptogenesis is the process of reactive astrogliosis (Burda and Sofroniew, 2014; Pekny and Pekna, 2016). This is a graded response to a wide array of insults, which is a hallmark of many neurological disorders (Burda and Sofroniew, 2014; Ferlazzo et al., 2016; Glushakov et al., 2016; Pekny and Pekna, 2016; Fordington and Manford, 2020; Galovic et al., 2021).

Reactive astrocytes are characterized by morphological and molecular changes (Figure 1). Specifically they proliferate, undergo hypertrophy and increase their expression of intermediary filament proteins like glial fibrillary acid protein (GFAP) and vimentin (Yang et al., 1994; Pekny and Nilsson, 2005; Sofroniew, 2009; Cregg et al., 2014; Escartin et al., 2021). *In extremis*, these changes may lead to the formation of a glial scar (Miller, 2005; Barres, 2008; Sofroniew, 2009; Burda and Sofroniew, 2014; Ferlazzo et al., 2016; Glushakov et al., 2016; Pekny and Pekna, 2016; Fordington and Manford, 2020; Galovic et al., 2021). Reactive astrogliosis can be observed in several acquired forms of epilepsy but has mostly been investigated in the context of mTLE (Wieser and ILAE Commission on Neurosurgery of Epilepsy., 2004; Blümcke et al., 2013; Cendes et al., 2014).

There is ample evidence that reactive astrocytes display aberrant Ca^2+^ signaling at least in the early phase of epileptogenesis (Table 1). Ding et al. (2007) found increased astrocytic Ca^2+^ activity in the days following pilocarpine-induced SE in mice. In the same study both *in vitro* and *in vivo* pharmacological approaches demonstrated that these Ca^2+^ signals could contribute to neuronal death, linking astrocytic hyperactivity to a key hallmark of epileptogenesis (Ding et al., 2007). We confirmed the astrocytic hyperactivity following SE by employing genetically encoded Ca^2+^ indicators in acute hippocampal slices from a mouse model of mTLE, and found that stimulation-evoked Ca^2+^ transients in astrocytic endfeet even outlasted those in cell bodies during the latent phase of epileptogenesis (Szokol et al., 2015).

Increased astrocytic Ca^2+^ activity has been anecdotally reported at even later time points after the initial insult (Enger et al., 2015; Shigetomi et al., 2019). These increased Ca^2+^ signals are likely stimuli- and stage specific and may reflect the degree of the reactive astrogliosis (Kuchibhotla et al., 2009; Fordsmann et al., 2019), as others have shown attenuated astrocytic Ca^2+^ activity in atrophic astrocytes in chronic epilepsy (Plata et al., 2018).

The degree, development and underlying mechanisms involved in aberrant Ca^2+^ signaling in epileptogenesis are still unknown, but it is plausible that several of the physiological signaling pathways involved in astrocytic Ca^2+^ dynamics (Caudal et al., 2020), could be perturbed. A major pathway for eliciting astrocytic Ca^2+^ signals is the activation of the Gq G-protein coupled receptors (GqPCRs) and subsequent release of Ca^2+^ from the endoplasmic reticulum via IP_3_R2 as discussed in “Astrocyte Ca^2 +^ signaling and Ictogenesis” (Figure 1; Foskett et al., 2007). Astrocytes express several GqPCRs, of which mGluR5 has attracted most attention due to an upregulation in epileptic tissue and potential involvement in an excitatory loop comprising glutamate induced Ca^2+^ dependent glutamate release from astrocytes (Umpierre et al., 2019). While astrocytes in the adult brain are almost depleted of mGluR5 (Sun et al., 2013), the receptor is consistently expressed in chronic epilepsy models and resected tissue from patients with epilepsy (Aronica et al., 2000, 2003), and a recent study has shown that mGluR5 expression and mGluR5-dependent Ca^2+^ transients reemerge during epileptogenesis along with an increase in glutamate uptake (Umpierre et al., 2019). This reemergence of astrocytic mGluR5 could potentially be a compensatory anti-epileptic mechanism to handle the elevated glutamate levels in epileptic tissue but could possibly also represent a pro-epileptic feature triggering downstream Ca^2+^ mediated gliotransmission.

Apart from these perturbations in glutamate dynamics, it has been shown that reactive astrocytes exhibit a tonic release of GABA, presumably through Bestrophin-1 channels (Pandit et al., 2020). Bestrophin-1 channels are Ca^2+^ activated anion channels, and increased GABA release could hence be a downstream effect of increased Ca^2+^ signaling in reactive astrocytes (Lee et al., 2010). In support of this conjecture is the finding of an accumulation of GABA in reactive astrocytes in a model of mTLE (Müller et al., 2020). Potentially, this is a protective aspect of reactive astrocytes to curb epileptiform activity in this pathological tissue.

Moreover, as mentioned in “Ictogenesis” astrocytic Ca^2+^ signaling has been suggested to be involved in homeostatic mechanisms of astrocytes. These mechanisms could be important downstream effects of astrocytic Ca^2+^ dyshomeostasis in epileptic tissue, but these effects are so far rudimentarily investigated in epilepsy.

Loss of astrocytic gap junction coupling has been shown to occur during early epileptogenesis in experimental models of mTLE and in specimens of resected hippocampi from patients with mTLE (Bedner et al., 2015; Deshpande et al., 2017, 2020; Henning et al., 2021). It is believed that this loss of astrocytic coupling in epilepsy may perturb the ability of astrocytes to remove K^+^ from the extracellular space through the process of K^+^ spatial buffering (Nwaobi et al., 2016). Notably, astrocytic gap junctions may also allow Ca^2+^ signals to propagate from cell to cell, at least during pathological conditions like seizure activity (Scemes and Giaume, 2006). It is tempting to hypothesize that such propagating Ca^2+^ waves could play a role in neuronal synchronization and seizure generation. Potentially a loss of astrocytic gap junctions as seen in epileptic tissue, may be a compensatory mechanism to prevent intercellular spread of astrocytic Ca^2+^ waves. Even so, to the best of our knowledge, no direct study of astrocytic Ca^2+^ signaling in gap junction deficient mice has been performed.

Loss of the highly concentrated expression of key membrane channels in astrocytic endfoot processes, i.e., loss of astrocyte polarization, is another pathological hallmark, which could be a consequence of perturbed glial Ca^2+^ dynamics (Figure 1). For instance AQP4 and Kir4.1 are normally densely expressed in astrocytic endfeet, kept in place by the so-called dystrophin associated protein complex (DAPC) (Nagelhus et al., 1998; Enger et al., 2012), and in tissue resectates from patients with mTLE, a striking loss of this polarized expression of both AQP4 and Kir4.1 have been shown (Eid et al., 2005; Heuser et al., 2012). It is possible that prolonged epileptic activity and increased Ca^2+^ signaling in astrocytic endfeet, as we demonstrated in Szokol et al. (2015), activate Ca^2+^ dependent proteases like calpain (Nagelhus and Ottersen, 2013), that shows affinity to dystrophin and could cleave the DAPC (Figure 1; Shields et al., 2000).

Even though the evidence is indirect, it has been suggested that this loss of astrocyte endfoot polarization could contribute to epileptogenesis and hyperexcitation (Binder et al., 2012; Binder and Carson, 2013; Crunelli et al., 2015). Notably, the loss of the astrocyte endfoot Kir4.1 channels in tissue from mTLE patients (Heuser et al., 2012) is expected to cause impaired K^+^ handling and resultant neuronal hyperexcitation due to the role of Kir4.1 in K^+^ homeostasis (Bordey and Sontheimer, 1998; Hinterkeuser et al., 2000; Kivi et al., 2000; Neusch et al., 2001; Djukic et al., 2007; Bockenhauer et al., 2009; Scholl et al., 2009; Steinhäuser et al., 2012).

## Conclusion and Future Perspectives

Here we have discussed the role of astrocyte Ca^2+^ signaling in *ictogenesis* and *epileptogenesis*. These terms are used to describe two different features of epilepsy, but do not necessarily imply two separate processes, as mechanisms crucial in ictogenesis could also be an integral part of epileptogenesis, or vice versa. While we often associate astrocytic dysfunction in *epileptogenesis* with the appearance of *reactive astrogliosis* (Escartin et al., 2021), the term *ictogenesis* seems typically to be used when studying the interplay between neurons and astrocytes independent of pre-existing tissue pathology. Therefore, we may overlook the fact that ictogenesis most often would occur in tissue that has undergone pathological transformation typical for *epileptogenesis*, i.e., not normal, healthy tissue. On the other hand, *epileptogenesis* comprises many pathological changes beyond *reactive astrogliosis*, like alterations in transcriptional regulation, morphological, biochemical, metabolic and physiological remodeling ultimately resulting in gain or loss of function (Escartin et al., 2021).

Astrocytic Ca^2+^ signals are today considered a main readout of astrocytic activity and there are reasons to believe that they play important roles in epilepsy. Evidence suggests that such signals are neither necessary nor sufficient to maintain epileptiform activity, but rather should be seen as modulators of the pathophysiological process. The literature directly investigating the role of astrocytic Ca^2+^ signaling in epilepsy is still sparse and at some points contradictory, and for most proposed mechanisms only a small subset of the signaling pathways involved are identified. A major challenge will be to disentangle the potentially beneficial from detrimental consequences of the different modes of astrocyte Ca^2+^ signaling in reactive astrogliosis. It is even probable that astrocyte Ca^2+^ signaling may carry different roles in the large variety of epileptic entities. To decipher the roles of astrocyte Ca^2+^ signaling in epilepsy, next steps should include a rigorous study of the mechanisms mentioned above *in vivo* in adult mice, leveraging new developments in both imaging and genetics, with the aim of identifying promising targets for future pharmacological therapy of epilepsy.

## Author Contributions

KH and RE reviewed the literature, conceptualized the manuscript, and wrote the manuscript. Both authors contributed to the article and approved the submitted version.

## Conflict of Interest

The authors declare that the research was conducted in the absence of any commercial or financial relationships that could be construed as a potential conflict of interest.
